# Identification of Potential Nematicidal Compounds against the Pine Wood Nematode, *Bursaphelenchus xylophilus* through an *In Silico* Approach

**DOI:** 10.3390/molecules23071828

**Published:** 2018-07-23

**Authors:** Gnanendra Shanmugam, Sun Keun Lee, Junhyun Jeon

**Affiliations:** 1 Department of Biotechnology, College of Life and Applied Sciences, Yeungnam University, Gyeongsan 38541, Korea; gnani.science@gmail.com; 2 Division of Forest Insect Pests and Diseases, National Institute of Forest Science, Seoul 02455, Korea; lskyou@korea.kr

**Keywords:** *Bursaphelenchus xylophilus*, pine wood nematode, nematicidal compounds, amocarzine, flubendazole, molecular docking, density functional theory

## Abstract

*Bursaphelenchus xylophilus* is a destructive phytophagous nematode that mainly infects pine species and causes pine wilt disease (PWD). PWD is one of the most devastating diseases that has damaged the pine forests of eastern Asia and Portugal for the last four decades. *B. xylophilus* infects healthy pine trees through *Monochamus* beetles and its subsequent proliferation results in destruction of the infected pine trees. The poor water solubility and high cost of currently used trunk-injected chemicals such as avermectin and abamectin for the prevention of PWD are major concerns. Thus, for the identification of new compounds targeting the different targets, five proteins including cathepsin L-like cystein proteinase, peroxiredoxins, hsp90, venome allergen protein and tubulin that are known to be important for development and pathogenicity of *B. xylophilus* were selected. The compounds were virtually screened against five proposed targets through molecular docking into hypothetical binding sites located in a homology-built protein model. Of the fifteen nematicides screened, amocarzine, mebendazole and flubendazole were judged to bind best. For these best docked compounds, structural and electronic properties were calculated through density functional theory studies. The results emphasize that these compounds could be potential lead compounds that can be further developed into nematicidal chemical against *B. xylophilus*. However, further studies are required to ascertain the nematicidal activity of these compounds against phytophagous nematode.

## 1. Introduction

The pine wood nematode *Bursaphelenchus xylophilus* is a migratory endoparasitic nematode causing pine wilt disease (PWD). This pine wood nematode (PWN) has caused severe damage to the forest ecosystems of Far East Asian countries [1] and North America [2]. Since its first report in Japan in 1905 [3], PWN has become an economically important pest throughout the East Asian countries [4]. Spread of this notorious invasive species to Portugal and Spain in Europe has posed the serious threat to the pine forests across the globe [5]. This phytoparasitic nematode can be transmitted from dead tree to healthy trees through pine sawyer beetle vectors (*Monochamus* spp.) [6]. Unlike other species in the *Bursaphelenchus* genus, *B. xylophilus*, which possess both phytophagus and mycophagus feeding strategies, are unique [7]. The nematode feeds on the parenchyma cells by migrating through resin canals and causes wilting of pine trees. Once the pine trees are dead, the nematode starts feeding on the fungi (*Botrytis cinerea*) that colonize the dead trees [8].

In general, the fumigation of affected trees with metam-sodium, control of Japanese sawyer beetle vectors (the only vector of PWN) with aerial application of insecticides (fenitrothion and thiacloprid) and the trunk injections of nematicides such as avermectin, abamectin, morantel tartarate and levamisole hydrochloride are currently practiced as control measures [9,10]. However, the poor solubility and higher cost are the greater concerns in the usages of trunk injections to control the PWN. Likewise in *Trichostrongylus colubriformis* and *Ostertagia circumcincta* resistance to ivermectin [11], in near future the concern about *B. xylophilus* developing resistance to avermectin, abamectin, morantel tartarate and levamisole hydrochloride due to their continuous usage may occur. However, PWN has not yet been reported to show resistance, there are several other pest insects that have developed resistance to emamectin benzoate, one of nematicidal compounds the most widely used as a trunk injection agent in China [12,13,14,15]. Thus the identification of novel targets, compounds that act as antinematodal agents and elucidating their mechanism of action might serve the purpose.

The availability of the completely sequenced genome of *B. xylophilus* has opened up the possibility of investigating the most prominent proteins that play a crucial role in the survival and parasitism of the nematode as novel drug targets [16,17]. Hence in this study, cathepsin L-like cysteine proteinase that is involved in post embryonic development [18], 2-cysteine peroxiredoxins that regulates reproduction and pathogenecity [19], heat shock protein 90 (HSP90) that helps adaptation to different climatic conditions [20], venom allergen proteins (VAP) that manifests the invasion of parasitic genes [21] and tubulin that regulates the microtubule, mitosis and motility [22,23] of *B. xylophilus* are considered as drug targets. In general, the anti-nematode drugs are classified into two main types based on their target sites, such as drugs targeting membrane ion channels and other class of drugs that acts on biochemical targets. Thus, in this present study, the compounds belonging to the benzimidazole, imidazothiazole and tetrahydropyrimidines compound classes that act on biochemical targets are selected for virtual screening. The virtual screening was carried out to reveal the better binding affinities against these targets as potential alternatives of PWN control agents. In addition, structural and electronic properties of best compounds were calculated through density functional theory (DFT) studies to explore the sites of chemical reactivity that are vital for the compounds for the possible interactions with receptors. The results emphasize that a nematicidal compound that binds to more than one drug target can be used to more effectively control PWD, instead of using the chemical agent that bind to single specific drug target.

## 2. Results and Discussion

In last few decades, virtual screening strategies such as molecular docking have made a significant impact on the discovery of promising new drug leads [24]. Many research groups have put forth the usage of these molecular docking methods to screen potential novel compounds against various diseases [25]. By employing similar approaches, eight phenylpropanoids were reported as inhibitors against a migratory endoparasitic nematode, *Radopholus similis*, which causes necrosis of plant tissues and massive destruction in host plants [26]. In the present study, we have explored the binding efficiencies of compounds with nematicidal activity through docking studies against five potential targets from *B. xylophilus* (Table 1)*.* All the compounds included in the study exhibited docking interactions with all five protein targets. However, amocarzine, flubendazole and mebendazole are considered to be the best in terms of their binding energies against all the five target proteins, when compared to the inactive antihelmenthic compound, chloramphenicol (see below).

### 2.1. Target–Template Alignment for Homology Modeling

The BLASTP [27] analysis of target sequence such as cathepsin L-like cysteine proteinase (BxCLCP), 2-cysteine peroxiredoxins (BxPRX), heat shock protein 90 (BxHSP90), venom allergen protein-3 (BxVAP-3) and tubulin protein (BxTUB) sequence against protein databank (PDB) [28] identified 1CS8, 1QMV, 5FWP, 4NUI and 5IJ0 X-ray crystal structures as homologous proteins respectively. These X-ray crystal structures shared sequence identity of 56.88%, 74.61%, 74.86%, 40.94% and 86.38% with the five target proteins, which significantly implies that they were functionally related. Hence these proteins were considered as templates for homology modelling. Further, these templates were analyzed for its resolution, sequence similarity and secondary structure similarity covering the maximum range of target sequence. Few studies have demonstrated that sequence identity higher than 25% between two proteins are similar in 3D structures [29,30]. Hence, the 3D structures of respective templates were considered to be suitable for homology modeling.

### 2.2. Homology Modeling

The template–target sequence alignment files were used to generate the 3D models of BxCLCP, BxPRX, BxHSP90, BxVAP-3 and BxTUB by using the template structure co-ordinates in homology modeling tool, Modeler9v9 [31].

**Figure 1:**
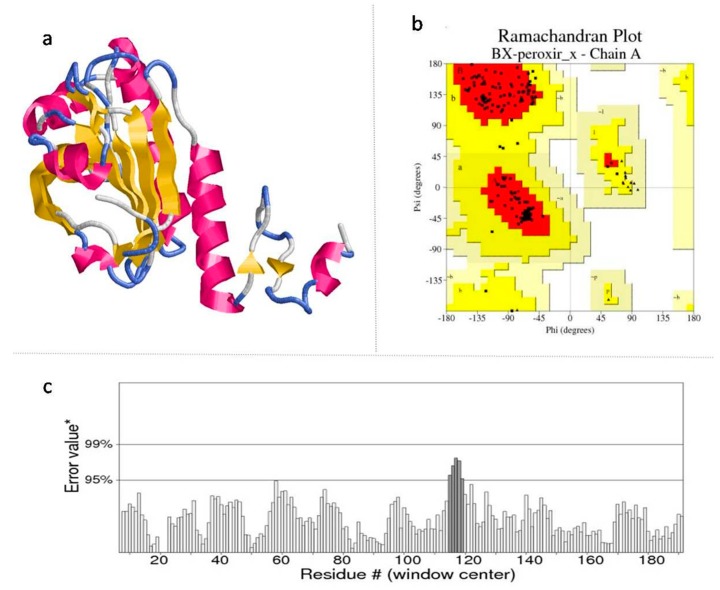
Theoretical model of 2-cysteine peroxiredoxin structure and model validation through SAVES server. (**a**) The 3D structure of built protein in cartoon representation and structure colors: Helices (magenta), Sheets (yellow) and turns/loops (blue); (**b**) Model validation by Ramachandran plot; (**c**) The ERRAT Plot shows that the generated model has high resolution since only a small stretch of 5 amino acids in the modelled structure needs to be rejected at a confidence level greater that 95%.

The Discrete Optimized Protein Energy (DOPE) score of the models revealed the structural compatibility [32]. These modelled structures of BxCLCP, BxTUB, BxVAP-3 and BxHSP90 (Supplementary Figures S1–S4) and BxPRX (Figure 1a) were considered for further analysis, as they exhibited the lowest DOPE assessment score and minimized energy.

### 2.3. Model Validation

The qualities of the modelled structures was assessed through online quality evaluation tools such as PROCHECK, ERRAT and Verify 3D, which are provided through Structural Analysis and Verification Server (SAVES) [33] of UCLA-DOE Lab. The phi and psi angles that explore the stereo-chemical parameters of the energy-minimized models were determined by using PROCHECK [34]; the 1D–3D structure compatibility of the best models by Verify 3D [35] and the regions of the modelled structure that can be rejected at the 95% and 99% confidence intervals were predicted through ERRAT programs [36]. The Ramachandran plot (RC plot) of all the generated models reveals that the built models are best as they exhibited more number of residues in the most favorable regions (Supplementary Table S1), while low number of residues are observed in disallowed region of RC plot (Figure 1b). Also, the ERRAT plot (Figure 1c) and Verify 3D measuring quality factor values supported that the built models are relevant, reliable and of good quality (Supplementary Table S1).

### 2.4. Structure-Based Virtual Screening

The modelled proteins were submitted to the CASTp server [37] to predict binding sites. From the predicted binding sites, the sites with larger volumes were used for further docking interaction with the anti-nematode compounds. Further, these binding pockets were assessed for docking with active (mebendazole) and in-active antihelmenthic compound (chloramphenicol). The pocket with higher binding affinity for active compound, mebendazole from each target was selected for further docking studies with 15 nematicidal compounds. Eventually, antihelmenthic compound with predicted molecular properties confined to the drug-like properties (based on Lipinski’s rule of five) and biological activity prediction (antihelmentics) at PASS (Predicted Activity Spectrum of Small Molecules) server [38] resulted in 15 compounds. These 15 FDA approved antihelmenthic compound were selected for further docking studies, as they can be easily used for drug repurposing and reformulation. The evaluation of drug-likeness of compounds is an essential part of drug discovery especially at the initial stages [39]. By considering the physicochemical properties of a compound using in silico approaches, its molecular impact in terms of in-vivo biological activity can be determined. The biological activity predicted at PASS server reveals the activities of compounds as active and inactive in its respective lab experiments. The biological activity spectrum at PASS server (Pa-Pi values) varies from 0.000 to 1.000. In PASS prediction, if the biological activity value of a compound is Pa > 0.5, then that compound is expected to reveal the activity in in-vivo and in-vitro experiments. If Pa > 0.7, then the compound is very likely to exhibit good activity in the experiments. The biological activity at PASS server is predicted for antihelminthic properties of all the 15 compounds (Supplementary Table S2). Further, the drug-like properties that satisfy the Lipinski’s rule of five, which is also considered to be essential for rational drug design, were also determined. It is observed that all the selected 15 compounds showed no violation of Lipinski’s rule of five [40] (Supplementary Table S3), i.e., ≤5 hydrogen bond donors, ≤10 hydrogen bond acceptors, <500 dal of molecular weight, <5 partition coefficient (log P), <10 rotatable bonds, topological polar surface area (TPSA) < 140.

To explore the binding efficacy and the molecular basis of interactions, the nematicidal compounds were docked within the predicted binding sites of all five modelled protein targets from *B. xylophilus*. All 15 nematicidal compounds exhibited docking poses with better binding affinities (in terms of docking score) against all five target proteins from *B. xylophilus* (Supplementary Table S3), when compared to inactive compound, chloramphenicol (BxCLCP: −1.0258 kJ/mol; BxPRX: −1.1087 kJ/mol; BxHSP90: −1.0122 kJ/mol; BxVAP-3: −1.1453 kJ/mol; BxTUB: −2.4862 kJ/mol). The binding energies and amino acids interactions of each compound against each target were given in Supplementary Tables S4–S8. From the docking results, it is evident that all the compounds exhibited binding energy against each targets.

The binding energies of all 15 compounds with five targets were given in Table 2. Although, all the compounds exhibited binding conformations in the active site pocket with both H-bond and non-bonded interaction, amocarzine, flubendazole and mebendazole exhibited better interaction (based on binding energies) with all five targets (Table 3).

The clear understanding of binding modes of these three compounds, amocarzine, flubendazole and mebendazole within the binding site of modelled structures can aid in the design of better nematicidal inhibitors against *B. xylophilus*. Thus, the docking interactions of amocarzine, flubendazole and mebendazole against all five proteins were analyzed in detail. The binding interactions of these compounds with all targets are stabilized with hydrogen bond and non-bond interactions (Supplementary Figures S5–S8). Generally, the non-bonded interaction is involved to make the protein-ligand complex more stable and establishes non-bonded force (vander waal’s) to make the ligands achieving its stable conformation for better activity [41].

In a similar study, Babu et al. [26] virtually screened the phenylpropanoids phytochemicals from *Piper nigrum* L. (black pepper) against various targets including β-1, 4, endoglucanase, cathepsin B-like cysteine proteinase and glutathione S-transferase from *Radopholus similis* and also reported the mortality rate through in-vitro assay. Sharma et al. [41] performed the docking interactions of nematicidal compounds with β-tubulin protein from *Brugia malayi* and reported albendazol sulfone as best nematicidal (anti-filarial) drug. Taylor et al. [22] has reported the common chokepoint reactions and enzymes in nematodes and prioritized the drug targets and suggested perhexiline as a nematicidal compounds and its binding efficacy against carnitine palmitoyltransferase 2 from *Caenorhabditis elegans*. In another study, Khanna and Ranganathan [17], reported benzimdazole and piperazine compounds as active compounds targeting acetylcholine and tubulin β-1 chain receptor from nematodes through in silico approach. They reported that piperazine-like substructures with nitrogen atom in the piperazine ring might be frequently involved in binding to the receptor.

In BxCLCP and BxTUB, flubendazole exhibited better binding energy (−19.3639 kJ/mol and −28.058 kJ/mol) followed by amocarzine (−18.7524 kJ/mol and −27.1220 kJ/mol) and mebendazole (−18.3215 kJ/mol and −25.531 kJ/mol). The binding interactions of flubendazole in BxCLCP are supported by H-bond formations with Ile25, Gln62, Cys65 and Thr206. The hydrophobic (non-bonded) interactions are supported by Gln62, Cys65, Gly66, Cys68, His207 and Trp230. The flubendazole interaction with BxTUB is supported by Gln11, Asn99, Gly142 and Thr143, while the non-bonded interactions are supported by Cys12, Asn99, Gly141, Asp177, Thr178, Asn204 and Tyr222.

**Figure 2:**
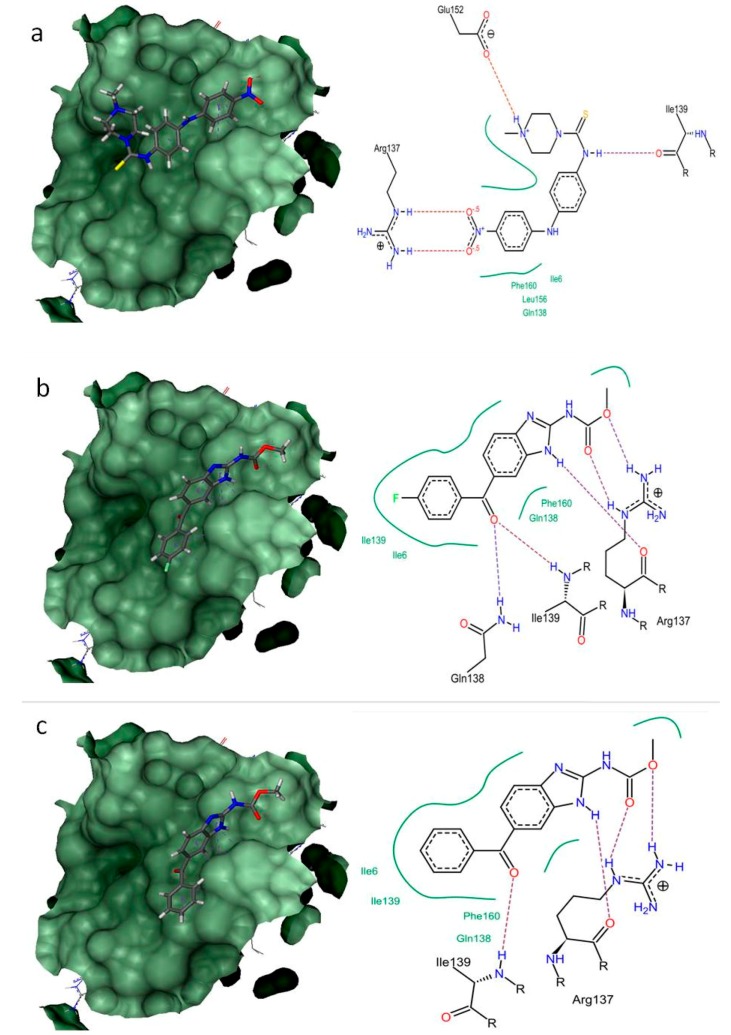
The docking complex and interactions of best docked compounds with 2-cysteine peroxiredoxin from *B. xylophilus*. (**a**) Amocarzine (binding energy: −30.1634 kJ/mol) (**b**) Flubendazole (binding energy: −23.2623 kJ/mol) (**c**) Mebendazole (binding energy: −20.1114 kJ/mol).

In BxPRX, amocarzine showed better activity with binding energy of −30.1634 kJ/mol, followed by flubendazole (−23.2623 kJ/mol) and mebendazole (−20.1114 kJ/mol) (Figure 2). The interactions of amocarzine are supported by H-bond formation with Arg137, Ile139 and Glu152. The non-bonded interactions are favoured by Ile6, Gln138, Leu156 and Phe160. Also, in BxHSP90 and BxVAP-3, amocarzine exhibited better binding energy (−22.8945 kJ/mol and −19.2792 kJ/mol) followed by mebendazole (−18.9934 kJ/mol and −18.6991 kJ/mol) and flubendazole (−15.0529 kJ/mol and −17.9623 kJ/mol). The binding interaction of amocarzine in BxHSP90 is favoured by H-bonds by Lys332 and Arg337. The hydrophobic interactions are supported by Lys332, Ala333, Gln334, Arg337, Asp338, Ser339 and Met342. The amocarzine interaction with BxVAP-3 is favoured by H-bond formation with Trp95, Pro96 and Asn160 and non-bonded interactions with Trp95, Pro96, His97 and Asn160.

Thus the present docking studies of amocarzine, flubendazole and mebendazole with chosen protein targets from *B. xylophilus* implies that the presence of keto groups (C=O) and amide groups (-CO-NH) as electronegative elements in the compound were crucial for interactions with binding site residues and for its nematicidal activity. Therefore, these prioritized drug targets and drug compounds may play a pivotal role in the development of new and urgently-needed nematicidal drugs against *B. xylophilus*.

### 2.5. Density Functional Theory Analysis

**Figure 3:**
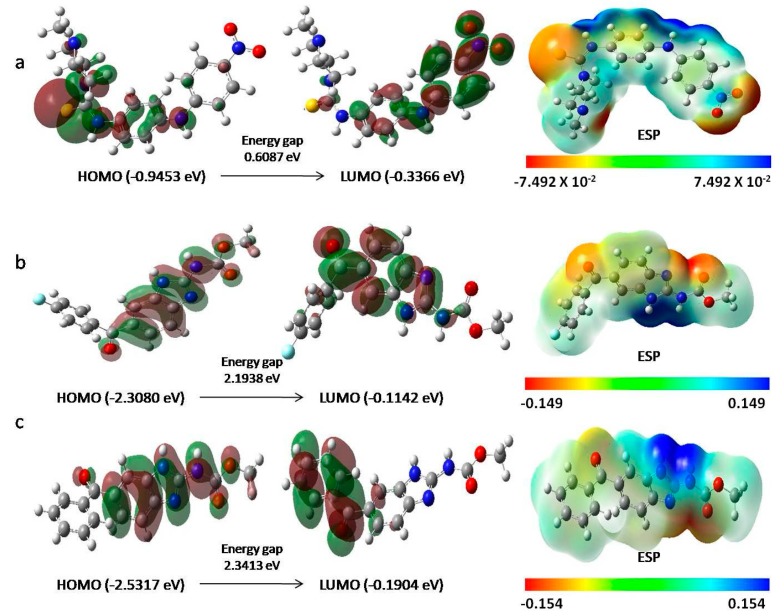
The HOMO-LUMO diagram and ESP maps of best docked compounds. (**a**) Amocarzine (**b**) Flubendazole (**c**) Mebendazole. The positive (red) and negative (green) phase distributions in molecular orbital wave function. HOMO (electron donor regions) determines the ionization potentials. LUMO (electron acceptor regions) determines the electron affinity. The ESP represents electron-rich (red) and electron-poor (blues) regions. The order of electron rich and poor regions are represented in rainbow colors as RED < ORANGE < YELLOW < GREEN < BLUE.

Density functional theory (DFT), a quantum mechanical approach is used to describe the accurate structural and electronic properties of compounds. In this study, the electronic distribution information of amocarzine, flubendazole and mebendazole were theoretically determined through orbital energy calculations. The idea on electronic distribution of these compounds could provide the clear picture of protein—ligand interactions and be useful to explore the binding modes of the compounds. The overall high and low electron density regions are better characterized by the electrostatic potentials (Figure 3). The Red and green colour distributions represent positive and negative phase in molecular orbital wave function, respectively. The localizations of the HOMO and LUMO of a ligand are very important because they influence the nature of the interaction with a proposed receptor. The HOMO of the ligand interacts with the LUMO of the receptor and vice versa. Hence, increasing the HOMO energy of the ligand closes the energy gap with the LUMO of the receptor and is expected to enhance binding. Similarly, lowering the LUMO energy of the ligand is expected to enhance binding. An electrostatic potential (ESP) map provides a picture of the overall polarity of a ligand [42].

The HOMO-LUMO diagram and ESP maps shows that the sulphur atom and nitrogen atoms in amocarzine contribute in HOMO and two nitro group oxygen atoms in LUMO (Figure 3a). In mebendazole, the HOMO is contributed by nitrogen and oxygen atoms, while LUMO is contributed by ring carbon atoms (Figure 3b). The nitrogen and oxygen atom along with ring carbon atoms in flubendazole contributes to HOMO and LUMO (Figure 3c). The HOMO orbital atoms are high electron density region that corresponds to functional groups interactions with the binding site residues within the target proteins as discussed above in structure-based virtual screening.

Among the screened compounds, the compounds that showed less HOMO-LUMO energy gap, low hardness and more softness are amocarzine (0.6087 eV, 0.3043 eV and 3.2862 eV), flubendazole (2.1938 eV, 1.0969 eV and 0.9116 eV) and mebendazole (2.3413 eV, 1.1706 eV and 0.8542 eV) (Supplementary Table S9). Thus the DFT calculations performed here further substantiate our molecular docking findings.

Eventually, to overcome the resistance development against the continuous usage of pesticides, the choice of novel compounds must have novel targets and different mode of actions. The target of currently used abamectin/avermectin is GABA-gated chloride channel [12]. Thus, the compounds targeting the different targets (biochemical targets) as reported in this study might be an alternative choice for the design of novel nematicidal compounds against *B. xylophilus.*

In conclusion, this study emphasize that amocarzine, mebendazole and flubendazole compounds can be used as potential lead compounds for the development of an effective nematicidal chemical against *B. xylophilus.* However their poor water-solubility can be overcome with cyclodextrins (hydroxypropyl-β-cyclodextrin) that are often used in the formulation of poor hydrosoluble compounds and in-vitro screening could result in a development of commercial trunk injection agent for PWD.

## 3. Materials and Methods

### 3.1. Sequence Analysis for Potential Templates

The five proteins targets, cathepsin L-like cystein proteinase (UniProt ID: Q6LDJ1), 2-cysteine peroxiredoxins (UniProt ID: B0LFQ7), HSP90 (UniProt ID: A4UU63), venom allergen proteins (UniProt ID: E0WW94) and tubulin (UniProt ID: D1MX18) protein sequence of *Bursaphelenchus xylophilus* was retrieved from UniProtKB database [43]. The most homologous sequence as potential template for homology modeling are obtained by using BLASTP (basic local alignment search tool) [27] similarity search tool against PDB database [28]. The sequence alignment and alignment errors were refined by using ClustalW [44] for homology modeling.

### 3.2. Homology Modeling

Using the homology modeling tool, Modeler9v9 [31], the homology models of five proteins (cathepsin L-like cysteine proteinase, 2-cysteine peroxiredoxins, HSP90, venom allergen proteins and tubulin) from *B. xylophilus* were built by employing the target–template sequence alignment files. A total of five 3D models for each of the target sequences were built from the starting structure of the templates by satisfying the spatial restraints through random generation [32]. Among the generated models, the least root mean square deviation (RMSD) value in comparison with template structure was considered for selecting the best model and its energy was minimized through 20 steps of steepest descent and conjugate gradient by using GROMOS [45] of Swiss-PDB viewer [46], and final energy-minimized model was used for further analysis.

### 3.3. Model Validation

The stereo-chemical parameters of the energy-minimized models were considered to evaluate the quality of the generated models. The phi and psi angles representing the stereo-chemical parameters of the model through PROCHECK [34], the compatibility of a generated 3D structure with its own amino acid sequence through Verify3D [35], and the regions of the modelled structure that can be rejected at the 95% and 99% confidence intervals through ERRAT [36] were determined at the SAVES server [33].

### 3.4. Structure-Based Virtual Screening

The binding pockets in the modelled structures were predicted by submitting to CASTp (Computed Atlas of Surface Topology of proteins) Server [37]. The binding sites with larger volumes and greater affinity towards active (mebendazole) antihelmenthic compound was selected for further docking studies. For virtual screening, the 15 antihelmenthic compound with predicted drug-like properties and biological activity (antihelmentics) from the ZINC database [47] were used. Docking was performed with 5 proteins of *B. xylophilus* by using FlexX [48]. The docking parameters were triangle matching base placements, full score (threshold 30) and No score contributions (threshold 70), 2.9 Å clash handling, 0.6 of protein ligand clashes and intra-ligand clash factors and 200 as maximum number of solutions per iteration.

### 3.5. Docking Interactions

The docking interactions of 15 compounds with the binding pocket amino acids of the each target protein was assessed by using pose-view [49], which clearly reveals the H-bond and non-bond interactions. The compounds with the better dock score and interaction were considered for further electronic structure studies.

### 3.6. Electronic Structure Study of Selected Screening Compounds

The molecular electrostatic potential (MEP), which determines polar interactions with the proposed binding sites, and the nature of the highest occupied and lowest unoccupied molecular orbital (HOMO and LUMOs), which determine nucleophilic and electrophilic activity, were calculated by using density functional theory (DFT). The DFT calculations were performed for amocarzine, flubendazole and mebendazole compounds by using functional B3LYP with 6-31G** basic set in Gaussian 09 [50]. The important parameter, HOMO-LUMO orbital energies that are used to assess the ionization energy, electron affinity [51], electro negativity, electronic chemical potential [52], molecular hardness, softness [53] and Electrophilicity index [54] are calculated to reveal the compounds stability and chemical reactivity.

## Figures and Tables

**Table 1:** The selected potential drug targets from *B. xylophilus* and its functions.

Sl. No.	Target	Function
1.	Cathepsin L-like cystein proteinase (BxCLCP) (UniProt ID: Q6LDJ1)	Post embryonic development
2.	2-cysteine peroxiredoxin (BxPRX) (UniProt ID: B0LFQ7)	Reproduction and pathogenecity
3.	Heat Shock Protein 90 (BxHSP90) (UniProt ID: A4UU63)	Adapts to different climatic conditions
4.	Venom allergen Protein-3 (BxVAP-3) (UniProt ID: E0WW94)	Invasion parasitic genes
5.	β-Tubulin (BxTUB) (UniProt ID: D1MX18)	Microtubule, mitosis, motility

**Table 2:** The binding energies (kJ/mol) of all compounds with all five target proteins from *B. xylophilus*.

Compound Name (Pubchem Id)	Cathepsin L-Like Cystein Proteinase (BxCLCP)	2-Cysteine Peroxiredoxin (BxPRX)	Heat Shock Protein 90 (BxHSP90)	Venom Allergen Protein-3 (BxVAP-3)	β-Tubulin (BxTUB)
Kainic acid (CID 10255)	−17.653	−18.586	−11.942	−12.681	−24.909
Carbendazim (CID 25429)	−14.879	−16.525	−12.365	−15.173	−21.44
Naphthalen-2-ol (CID 8663)	−9.5734	−11.793	−9.6124	−11.458	−12.053
Pyrantel (CID 708857)	−8.0396	−12.548	−8.519	−9.0794	−12.559
Closantel (CID 42574)	−15.618	−6.0856	−8.3846	−15.835	−14.155
Thiabendazole (CID 5430)	−12.071	−15.395	−12.1	−13.143	−16.532
Schaftoside (CID 442658)	−10.435	−9.6139	−4.0356	−5.3029	−22.876
Mebendazole (CID 4030)	−18.322	−20.111	−18.993	−18.699	−25.531
Oxfendazole (CID 40854)	−15.653	−19.8592	−13.344	−17.071	−21.242
Levamisole (CID 26879)	−8.1927	−12.361	−6.1674	−12.326	−13.724
Tetramizole (CID 3913)	−6.7261	−10.75	−5.2535	−12.811	−17.188
Coumafos (CID 2871)	−6.7703	−18.175	−1.7963	−5.8927	−15.065
Amocarzine (CID 5464102)	−18.752	−30.163	−22.895	−19.279	−27.122
Fenbendazole (CID 3334)	−14.391	−18.826	−14.743	−14.202	−24.141
Flubendazole (CID 35802)	−19.364	−23.2623	−15.053	−17.962	−28.058

**Table 3:** Amino acids in the binding pockets of the targets proteins favoring H-bond and non-bonded interactions with best docked compounds: Amocarzine, flubendazole and Mebendazole.

Potential Targets from *B. xylophilus*	Best Docked Compounds		
	Amocarzine (CID 5464102)	Flubendazole (CID 35802)	Mebendazole (CID 4030)
Cathepsin L-like cystein proteinase (BxCLCP) (UniProt ID: Q6LDJ1)	#Gln26 *, His27 #Glu28 *, Lys113 Thr206 *	Ile25 *, #Gln62 * #Cys65 *, Gly66 Cys68, Thr206 * His207, Trp230	#Gln62 *, #Cys65 *, Gly66, #Trp230 *
	−18.752	−19.364	−18.322
2-cysteine peroxiredoxin (BxPRX) (UniProt ID: B0LFQ7)	Ile6, Arg137 * Gln138, Ile139 * Leu156, Glu152 * Phe160	Ile6, Arg137 * #Gln138 *, #Ile139 *, Phe160	Ile6, Arg137 * Gln138, #Ile139 * Phe160
	−30.163	−23.2623	−20.111
Heat Shock Protein 90 (BxHSP90) (UniProt ID: A4UU63)	#Lys332 *, Ala333 Gln334, #Arg337 * Asp338, Ser339 Met342	#Met331*, Lys332 #Gln334 *, Ala335, #Arg337 *	Met331 *, Lys332 Ala333, Gln334* Ala335, #Arg337 *
	−22.895	−15.053	−18.993
Venom allergen Protein-3 (BxVAP-3) (UniProt ID: E0WW94)	#Trp95 *, #Pro96 * His97, #Asn160 *	Ala93, Gln94 #Trp95 *, #Asn160 *, Trp161	Ala93, Gln94 #Trp95 *, #Asn160 * Trp161
	−19.279	−17.962	−18.699
β-Tubulin (BxTUB) (UniProt ID: D1MX18)	Gln11, Gly98 * #Asn99 *, Ser138 Gly141, Thr143 * Ser176, #Asp177 * Glu181, Asn204	Gln11 *, Cys12 #Asn99 *, Gly141 Gly142 *, Thr143 * Asp177, Thr178 Asn204, Tyr222	Gln11, #Cys12 * Ser138, Gly141 Val169, Ser172 * #Asp177 *, Asn204 * Tyr222
	−27.122	−28.058	−25.531

* Residues involved in H-bond interactions; #* Residues involved in H-bond and non-bonded interactions. The other residues are involved in non-bonded interactions. Binding energies (kJ/mol) are provided respectively.

## Supplementary Materials

The following are available online, Figure S1: Theoretical model of Cathepsin L-Like cysteine proteinase (BxCLCP) structure and model validation through SAVES server; Figure S2: Theoretical model of Heat Shock Protein 90 (BxHSP90) structure and model validation through SAVES server; Figure S3: Theoretical model of Venom allergen Protein-3 (BxVAP-3) structure and model validation through SAVES server; Figure S4: Theoretical model of β-Tubulin (BxTUB) structure and model validation through SAVES server; Figure S5: The docking complex and interactions of best docked compounds with Cathepsin L-Like cysteine proteinase (BxCLCP) from *B. xylophilus*; Figure S6: The docking complex and interactions of best docked compounds with Heat Shock Protein 90 (BxHSP90) from *B. xylophilus;* Figure S7: The docking complex and interactions of best docked compounds with Venom allergen Protein-3 (BxVAP-3) from *B. xylophilus;* Figure S8: The docking complex and interactions of best docked compounds with β-Tubulin (BxTUB) from *B. xylophilus*; Table S1: The measured quality factor values along with ramachandrn plot residue distributions for the modelled proteins; Table S2: The predicted molecular properties confined to the druglike properties (based on Lipinski’s rule of five) and biological activity prediction (antihelmentics); Table S3: The 15 compounds with nematicidal activity considered in this study for docking against five targets from *B. xylophilus*; Table S4: Docking interactions of the binding site residues with all the 15 compounds against Cathepsin L-Like cysteine proteinase (BxCLCP) from *B. xylophilus*; Table S5: Docking interactions of the binding site residues with all the 15 compounds against 2-cysteine peroxiredoxin (BxPRX) from *B. xylophilus;* Table S6: Docking interactions of the binding site residues with all the 15 compounds against Heat Shock Protein 90 (BxHSP90) from *B. xylophilus;* Table S7: Docking interactions of the binding site residues with all the 15 compounds against Venom allergen Protein-3 (BxVAP-3) from *B. xylophilus;* Table S8: Docking interactions of the binding site residues with all the 15 compounds against β-Tubulin (BxTUB) from *B. xylophilus;* Table S9: DFT calculations pertaining to the molecular chemical reactivity for Amocarzine, flubendazole and Mebendazole.
