# Supplementary material for: Identification of Potential Nematicidal Compounds against the Pine Wood Nematode, Bursaphelenchus xylophilus through an In Silico Approach
Source: Molecules. 2018 Jul 23;23(7):1828. doi: 10.3390/molecules23071828 (PMC6100573; doi:10.3390/molecules23071828)
Supplement: Supplementary file 1 [file molecules-23-01828-s001.pdf]

Supplementary Data

Identification of potential nematicidal compounds against the pine wood nematode, Bursaphelenchus xylophilus through in silico approach

Gnanendra Shanmugam[1], Sun Keun Lee[2] and Junhyun Jeon[1]*

[1]Department of Biotechnology, College of Life and Applied Sciences, Yeungnam University, Gyeongsan, Gyeongbuk, 38541, Korea; gnani.science@gmail.com

[2]Division of Forest Insect Pests and Diseases, National Institute of Forest Science, Seoul 02455, Korea; lskyou@korea.kr

* Correspondence to Junhyun Jeon

Phone: +82 2 810-3030

E-mail: jjeon@yu.ac.kr

Supplementary Figures

Supplementary Figure S1

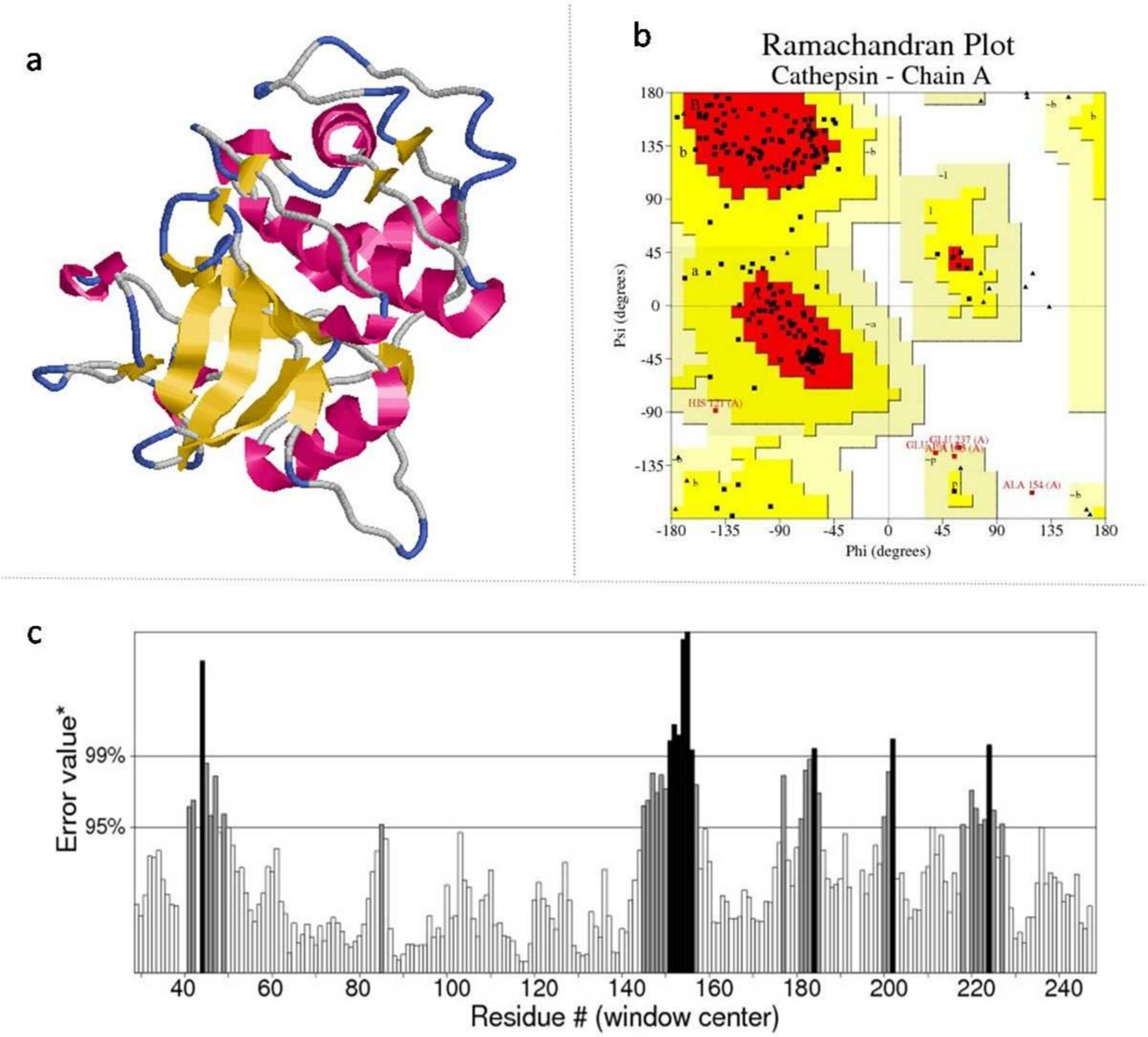

Theoretical model of Cathepsin L- Like cysteine proteinase (BxCLCP) structure and model validation through SAVES server. a. The 3D structure of built protein in cartoon representation and structure colors: Helices (magenta), Sheets(yellow) and turns /loops (blue). b. Model validation by Ramachandran plot. c. ERRAT Plot showing the generated model as good high resolution as the regions of the modeled structure that can be rejected at the 95% and 99% of confidence is very low.

Supplementary Figure S2

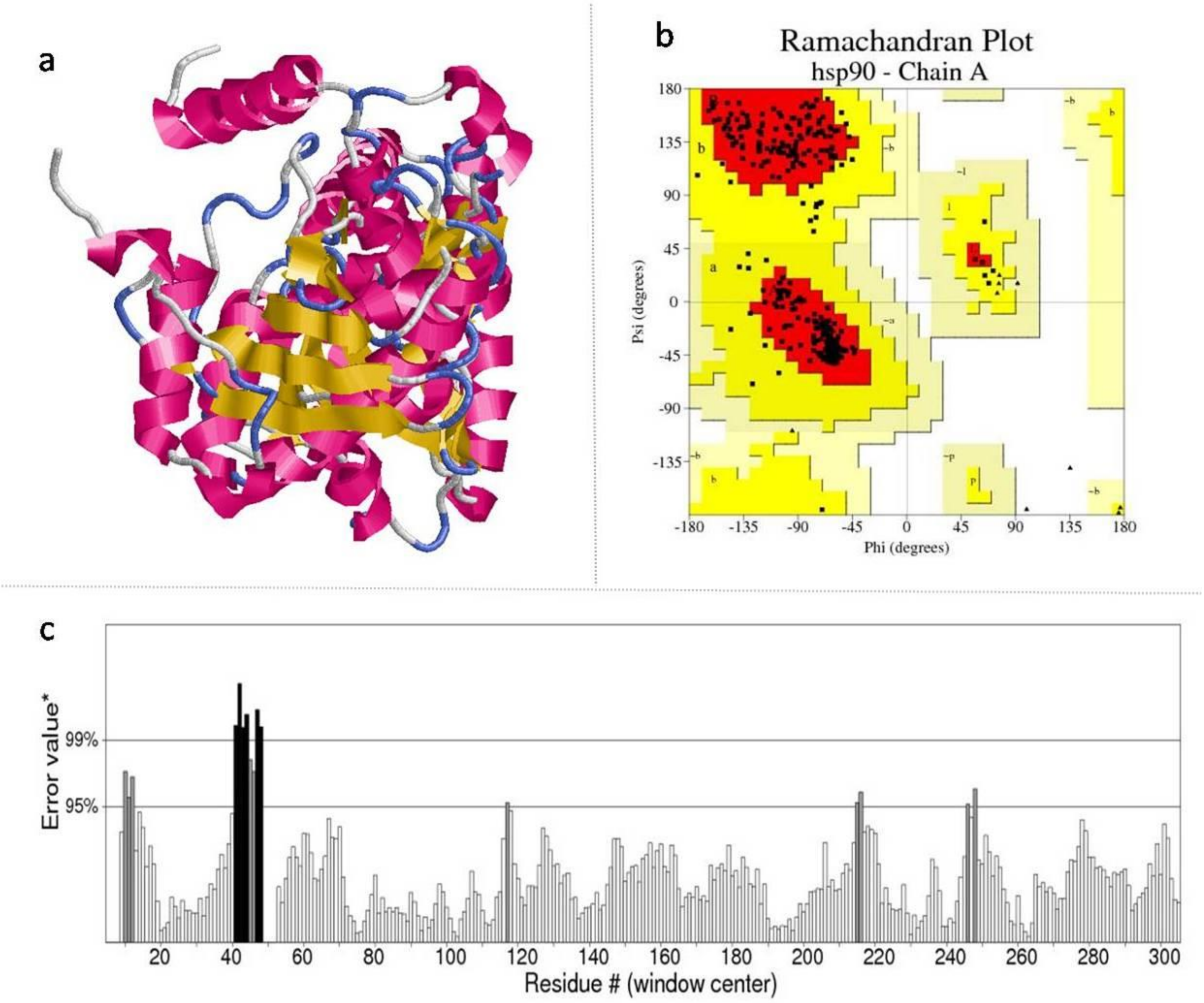

Theoretical model of Heat Shock Protein 90 (BxHSP90) structure and model validation through SAVES server. a. The 3D structure of built protein in cartoon representation and structure colors: Helices (magenta), Sheets(yellow) and turns /loops (blue). b. Model validation by Ramachandran plot. c. ERRAT Plot showing the generated model as good high resolution as the regions of the modeled structure that can be rejected at the 95% and 99% of confidence is very low.

Supplementary Figure S3

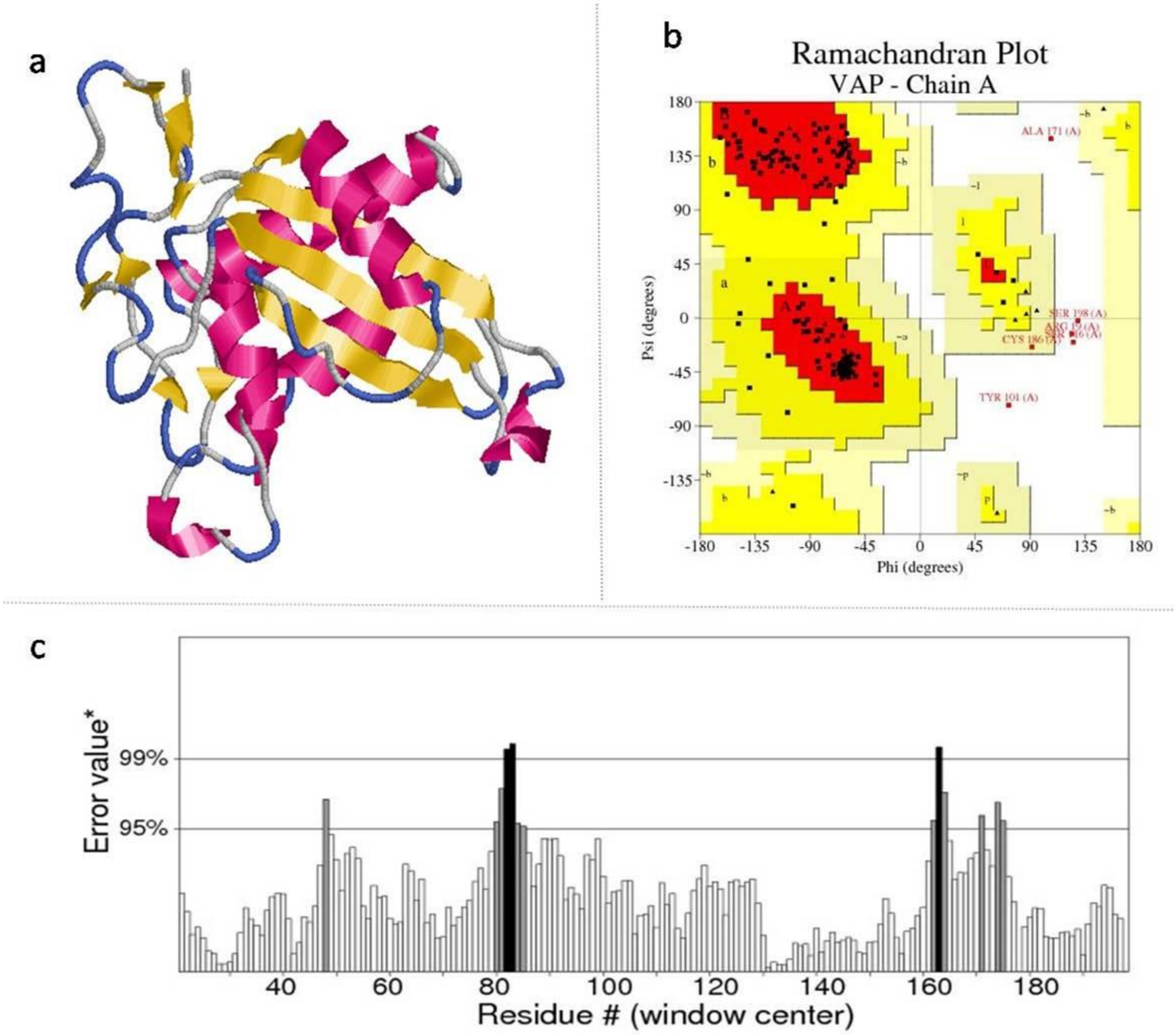

Theoretical model of Venom allergen Protein-3 (BxVAP-3) structure and model validation through SAVES server. a. The 3D structure of built protein in cartoon representation and structure colors: Helices (magenta), Sheets(yellow) and turns /loops (blue). b. Model validation by Ramachandran plot. c. ERRAT Plot showing the generated model as good high resolution as the regions of the modeled structure that can be rejected at the 95% and 99% of confidence is very low.

Supplementary Figure S4

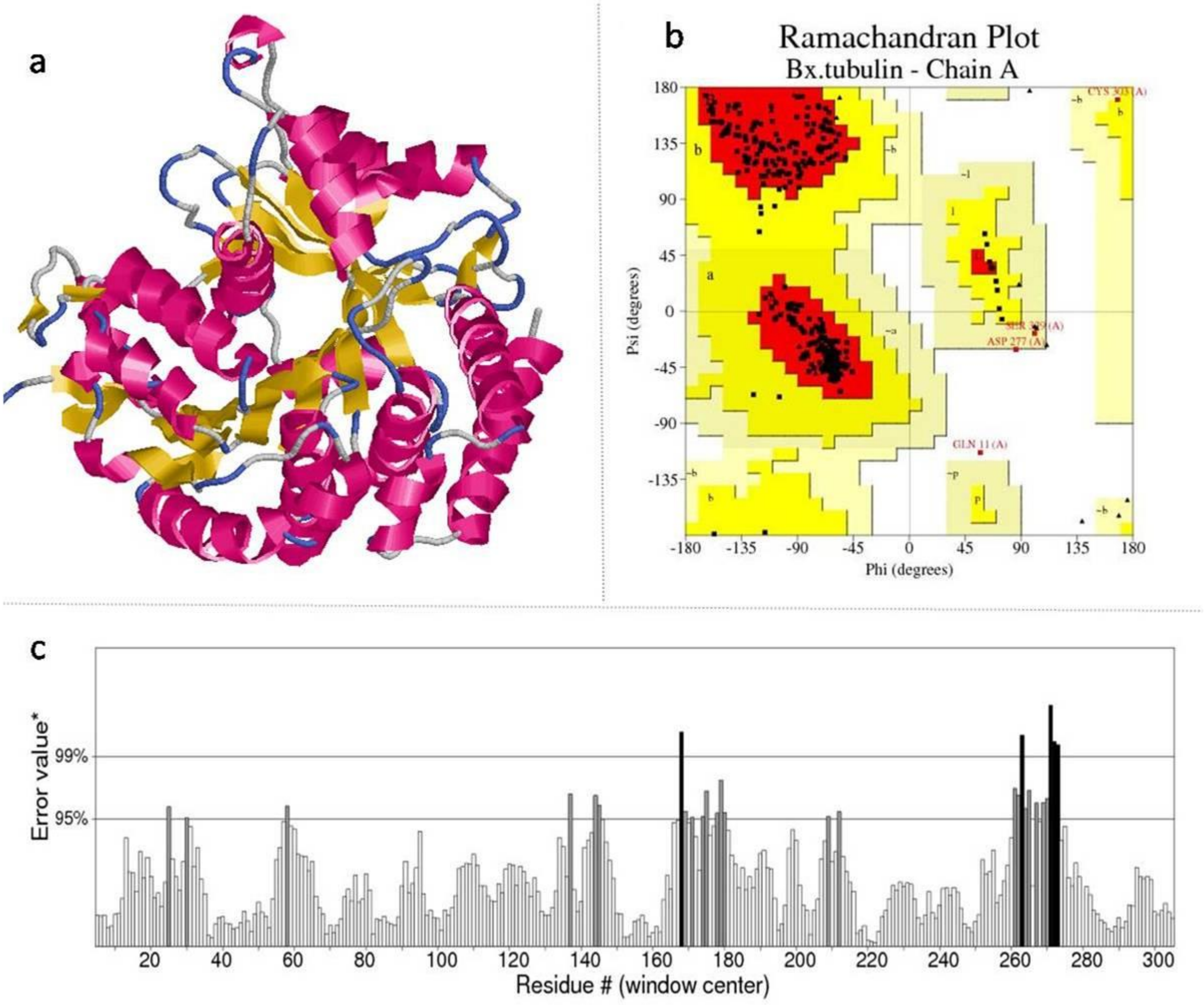

Theoretical model of β-Tubulin (BxTUB) structure and model validation through SAVES server. a. The 3D structure of built protein in cartoon representation and structure colors: Helices (magenta), Sheets(yellow) and turns /loops (blue). b. Model validation by Ramachandran plot. c. ERRAT Plot showing the generated model as good high resolution as the regions of the modeled structure that can be rejected at the 95% and 99% of confidence is very low.

Supplementary Figure S5

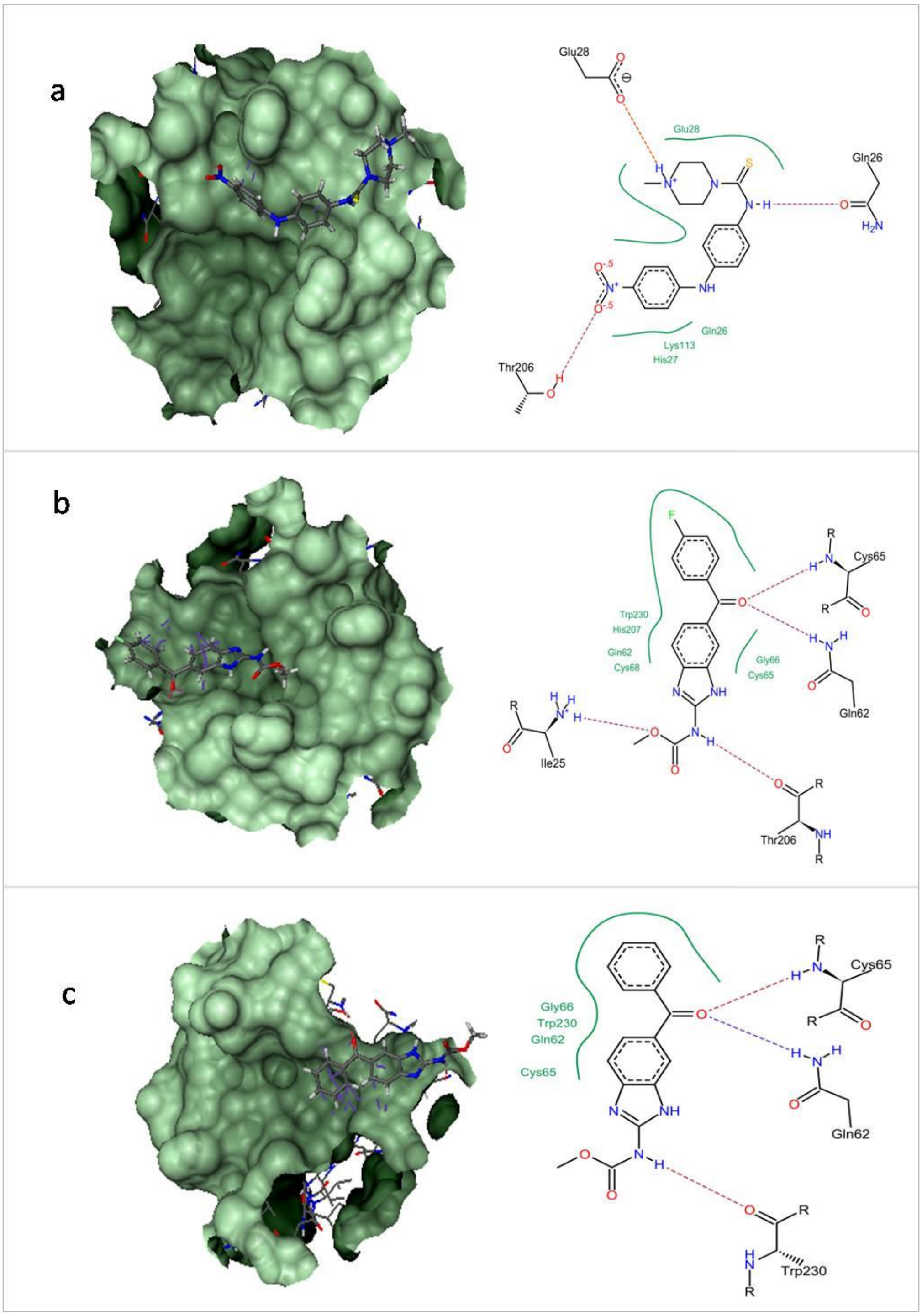

The docking complex and interactions of best docked compounds with Cathepsin L- Like cysteine proteinase (BxCLCP) from *B. xylophilus* . a. Amocarzine (Binding energy: -18.752 kJ/mol) b. Flubendazole (binding energy: -19.364 kJ/mol) c. Mebendazole (binding energy : -18.322 kJ/mol)

Supplementary Figure S6

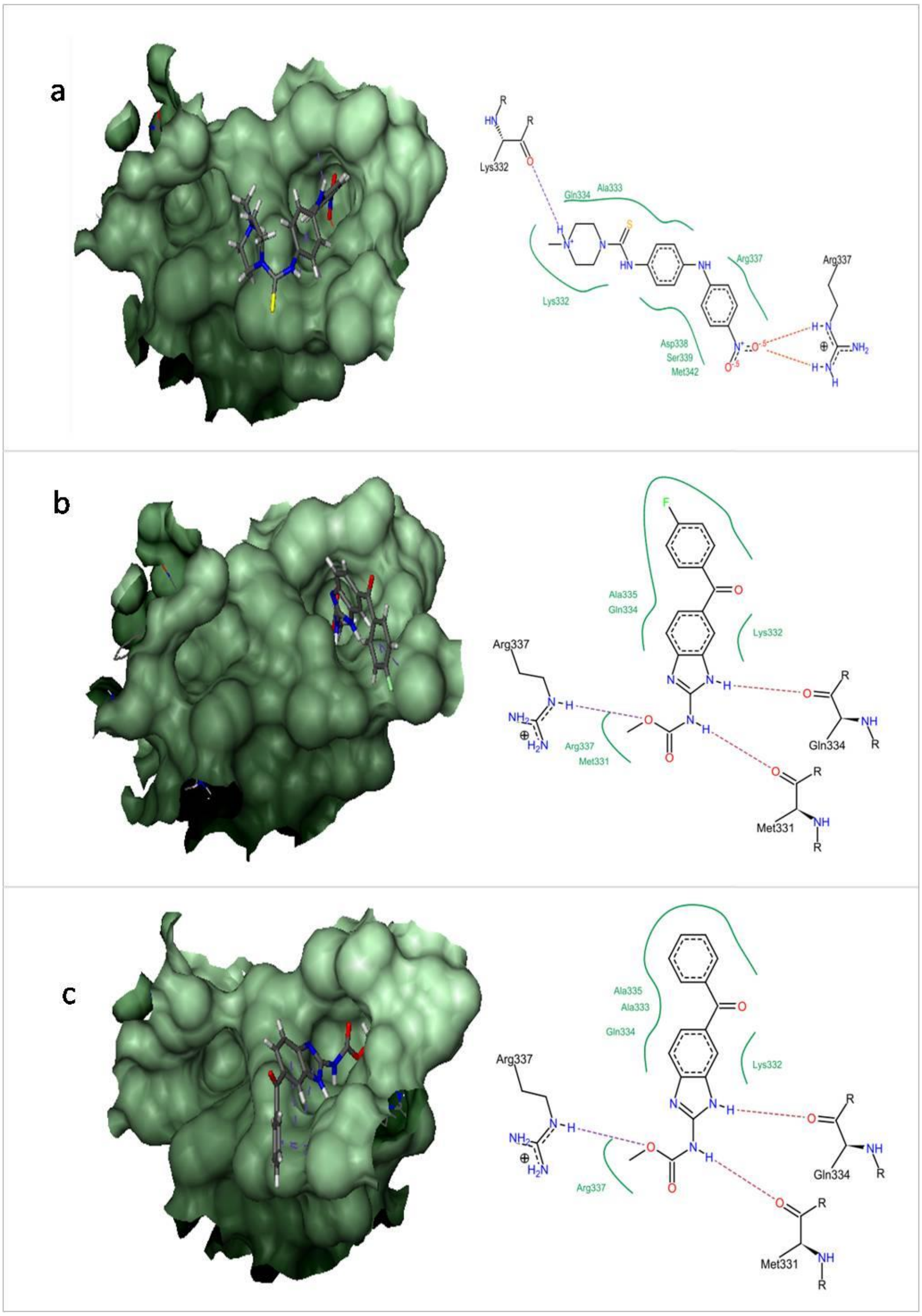

The docking complex and interactions of best docked compounds with Heat Shock Protein 90 (BxHSP90) from *B. xylophilus*. a. Amocarzine (Binding energy: -22.895 kJ/mol) b. Flubendazole (binding energy: -15.053 kJ/mol) c. Mebendazole (binding energy : -18.993 kJ/mol)

Supplementary Figure S7

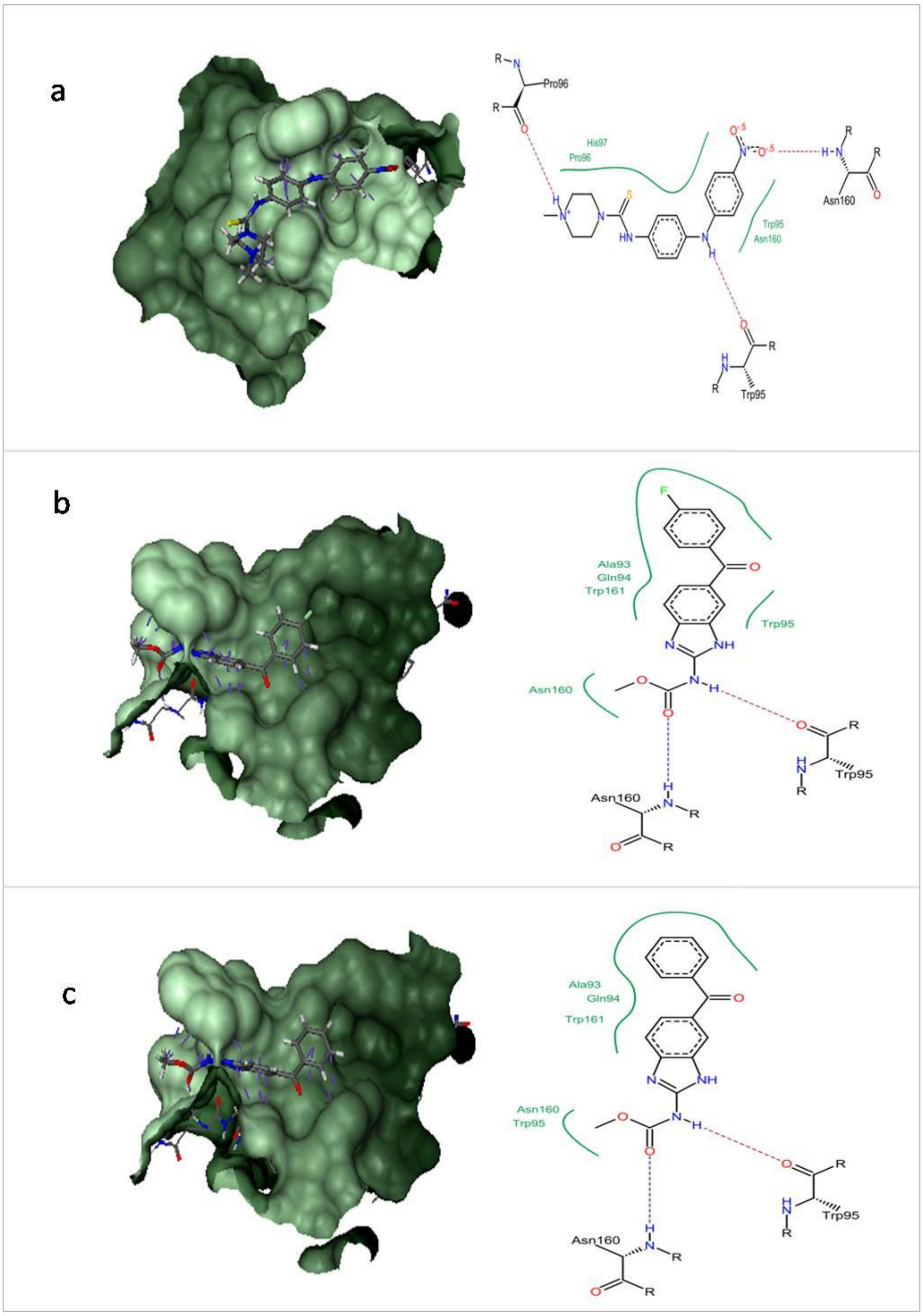

The docking complex and interactions of best docked compounds with Venom allergen Protein-3 (BxVAP-3) from *B. xylophilus* . a. Amocarzine (Binding energy: -19.279 kJ/mol) b. Flubendazole (binding energy: -17.962 kJ/mol) c. Mebendazole (binding energy : -18.699 kJ/mol)

Supplementary Figure S8

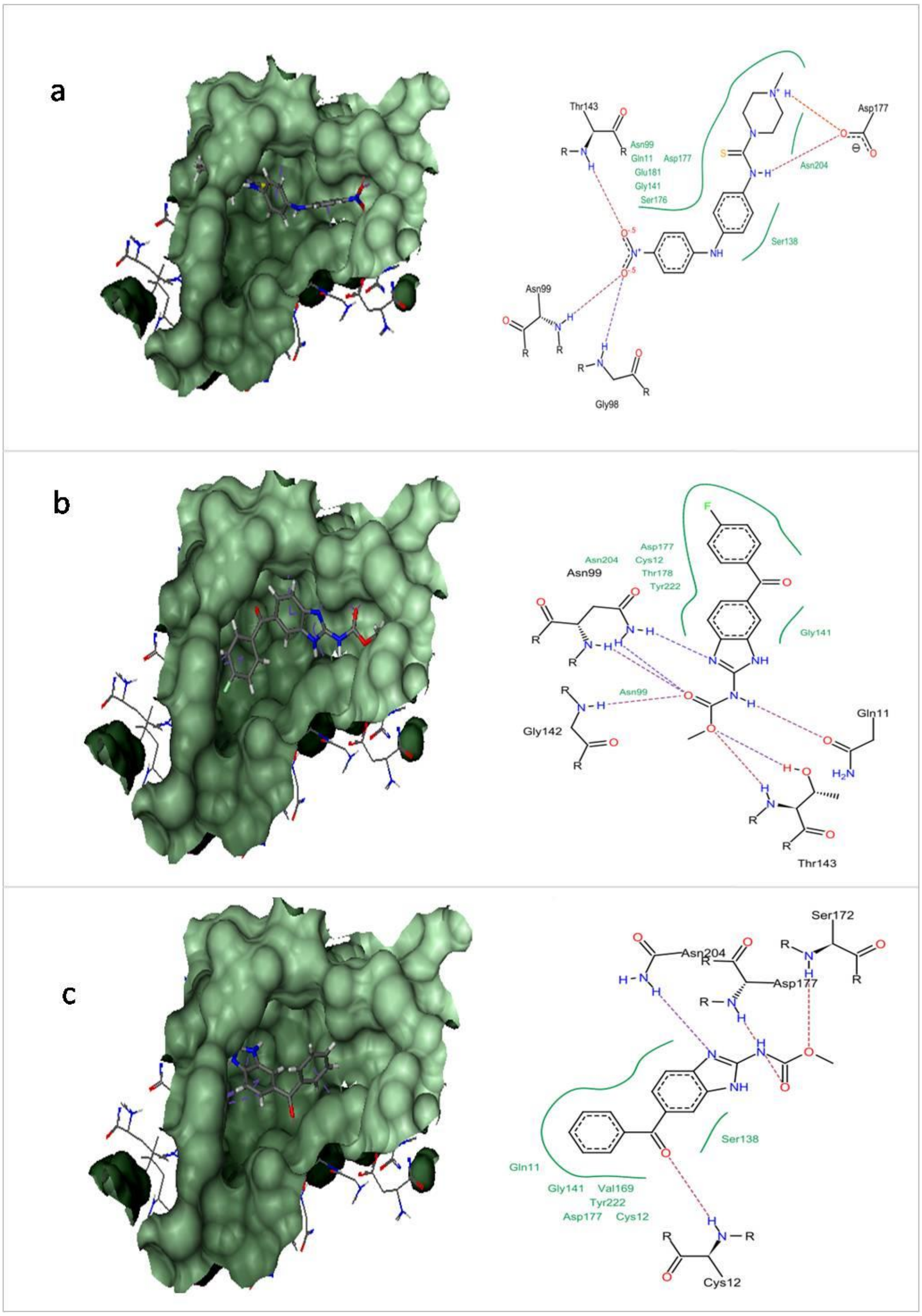

The docking complex and interactions of best docked compounds with β-Tubulin (BxTUB) from *B. xylophilus*. a. Amocarzine (Binding energy: -27.122 kJ/mol) b. Flubendazole (binding energy: -28.058 kJ/mol) c. Mebendazole (binding energy : -25.531 kJ/mol)

Supplementary Tables

Supplementary Table S1. The measured quality factor values along with ramachandrn plot residue distributions for the modelled proteins

S.No.	Model	Amino acids residues (%) in Ramachandran plot (PROCHECK)				Verify 3D (%)	ERRAT
		MFA	GAR	AAR	DAR		
1	BxCLCP	94.5	5.5	0.0	0.0	87.05	97.238
2	BxPRX	81.3	16.1	2.1	0.5	83.54	92.160
3	BxHSP90	92.8	7.2	0.0	0.0	90.38	95.750
4	BxVAP-3	85.7	10.6	0.6	3.1	89.78	92.164
5	BxTUB	94.7	4.2	0.5	0.5	98.83	93.258

MFA- Most favoured region; GAR-Generously allowed region; AAR- Additionally allowed region; DAR-Disallowed region.

Supplementary Table S2. The predicted molecular properties confined to the druglike properties (based on Lipinski's rule of five) and biological activity prediction (antihelmentics).

Compounds Pubchem ID	Molinspiration server predictions									PASS server predictions	
	miLogP	TPSA	natoms	Mol.Wt	nON	nOHNH	nviol	nrotb	volume	pa	pi
10255	-0.81	86.62	15	213.23	5	3	0	4	196.58	0.581	0.004
25429	1.46	67.02	14	191.19	5	2	0	2	165.8	0.882	0.002
8663	2.64	20.23	11	144.17	1	1	0	0	136.05	0.552	0.01
708857	2.47	15.6	14	206.31	2	0	0	2	194.34	0.78	0.003
42574	8.17	73.12	30	663.08	4	2	2	4	391.56	0.488	0.019
5430	2.35	41.58	14	201.25	3	1	0	1	166.83	0.862	0.002
442658	-1.68	250.96	40	564.5	14	10	3	4	461.51	0.343	0.072
4030	2.89	84.09	22	295.3	6	2	0	4	256.19	0.821	0.002
40854	2.84	84.09	22	315.35	6	2	0	4	262.88	0.902	0.001
26879	2.08	15.6	14	204.3	2	0	0	1	183.77	0.818	0.002
3913	2.08	15.6	14	204.3	2	0	0	1	183.77	0.818	0.002
2871	4.84	57.91	22	362.77	5	0	0	6	289.59	0.841	0.002
5464102	3.44	76.35	26	371.47	7	2	0	6	329.95	0.626	0.004
3334	3.4	67.02	21	299.36	5	2	0	4	255.33	0.914	0.001
35802	3.05	84.09	23	313.29	6	2	0	4	261.12	0.848	0.002

The molecular properties for the compounds are predicted at molinspiration server to define the compounds druglikness. miLogP-Molinspiration LogP (Octanol-water partition coefficient); TPSA- Total polar surface area (drug transport properties); natoms-Number of atoms; Mol.Wt (g/mol)- Molecular weight, nON- number of hydrogen bond acceptors; nOHHN- number of hydrogen bond donors; nviolations-Number of Lipinski's rule of five parameters violations; nrotb-Number of Rotatable Bonds (molecular flexibility); The biological activity at PASS server is predicted for antihemlminthic properties of the compounds. pa-probability to be active; pi-probability to be inactive.

Supplementary Table S3. The 15 compounds with nematicidal activity considered in this study for docking against five targets from *B. xylophilus*.

S.no	Pubchem id	Compound name	2D structure	SMILES notation
1.	10255	Kainic acid	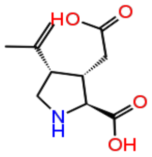	CC(=C)[C@H]1CN[C@@H]([C@H]1CC(=O)O)C(=O)O
2.	25429	Carbendazole	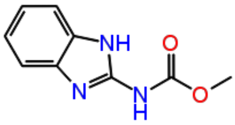	COC(=O)NC1=NC2=CC=CC=C2N1
3.	8663	2-Naphthol	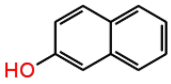	C1=CC=C2C=C(C=CC2=C1)O
4.	708857	Pyrantel	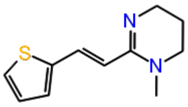	CN1CCCN=C1/C=C/C2=CC=CS2
5.	42574	Closantel	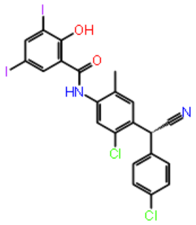	CC1=CC(=C(C=C1NC(=O)C2=CC(=CC(=C2O)I)I)Cl)C(C#N)C3=CC=C(C=C3)Cl
6.	5430	Thiabendazole	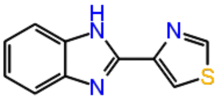	C1=CC=C2C(=C1)NC(=N2)C3=CSC=N3
7.	442658	Schaftoside	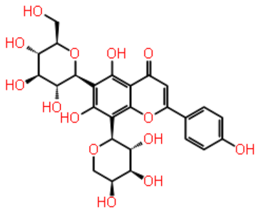	C1[C@@H]([C@@H]([C@H]([C@@H](O1)C2=C(C(=C(C3=C2OC(=CC3=O)C4=CC=C(C=C4)O)O)[C@H]5[C@@H]([C@H]([C@@H]([C@H](O5)CO)O)O)O)O)O)O)O

8.	4030	Mebendazole	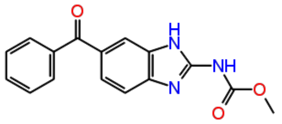	COC(=O)NC1=NC2=C(N1)C=C(C=C2)C(=O)C3=CC=CC=C3
9.	40854	Oxfendazole	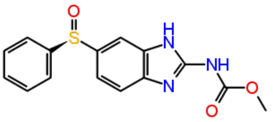	COC(=O)NC1=NC2=C(N1)C=C(C=C2)S(=O)C3=CC=CC=C3
10.	26879	Levamisole	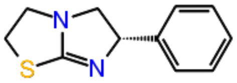	C1CSC2=N[C@H](CN21)C3=CC=CC=C3
11.	3913	Tetramisole	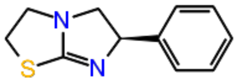	C1CSC2=NC(CN21)C3=CC=CC=C3
12.	2871	Coumaphos	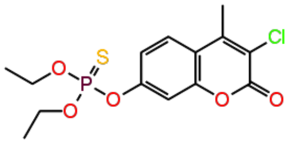	CCOP(=S)(OCC)OC1=CC2=C(C=C1)C(=C(C(=O)O2)Cl)C
13.	5464102	Amocarzine	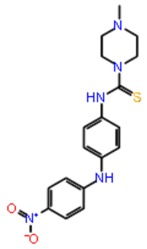	CN1CCN(CC1)C(=S)NC2=CC=C(C=C2)NC3=CC=C(C=C3)[N+](=O)[O-]
14.	3334	Fenbendazole	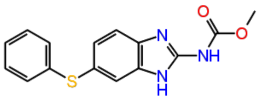	COC(=O)NC1=NC2=C(N1)C=C(C=C2)SC3=CC=CC=C3
15.	35802	Flubendazole	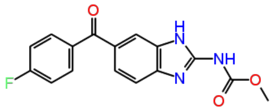	COC(=O)NC1=NC2=C(N1)C=C(C=C2)C(=O)C3=CC=C(C=C3)F

Supplementary Table S4. Docking interactions of the binding site residues with all the 15 compounds against Cathepsin L- Like cysteine proteinase (BxCLCP) from *B.xylophilus*

**10255**	**25429**	**8663**	**708857**	**42574**	**5430**	**442658**	**4030**	**40854**	**26879**	**3913**	**2871**	**5464102**	**3334**	**35802**
Ile25*	#Ile25*	Ile25	-	Ile25*	#Ile25*	#Ile25*	-	-	#Ile25*	-	#Ile25*	-	#Ile25*	Ile25*
-	-	-	Gln26	-	-	#Gln26*	-	-	-	-	-	#Gln26*	-	-
-	His27	-	-	-	His27	-	-	-	-	-	-	His27	His27	-
-	-	-	#Glu28*	-	-	-	-	-	-	-	-	#Glu28*	-	-
Gln62*	-	-	-	Gln62*	-	Gln62*	#Gln62*	#Gln62*	#Gln62*	Gln62*	Gln62*	-	-	#Gln62*
-	-	-	-	#Cys65*	-	-	#Cys65*	Cys65*	-	-	-	-	-	#Cys65*
Gly66	-	-	-	Gly66	-	Gly66	Gly66	-	Gly66	Gly66	-	-	-	Gly66
#Cys68*	-	-	-	-	-	Cys68	-	-	Cys68	Cys68	Cys68	-	-	Cys68
-	-	-	-	-	-	Asn104*	-	-	-	-	-	-	-	-
-	-	-	-	Asn107	-	#Asn107*	-	-	-	-	-	-	-	-
-	-	-	-	Gly108	-	Gly108	-	-	-	-	-	-	-	-
-	-	Asp110*	-	-	-	-	-	-	-	-	-	-	-	-
-	-	#Asp111*	-	-	-	-	-	-	-	-	-	-	-	-
-	-	-	Lys113	-	-	-	-	-	-	-	-	Lys113	-	-
-	-	Val176	-	-	-	-	-	-	-	-	-	-	-	-
-	-	Gly177	-	-	-	-	-	-	-	-	-	-	-	-
-	-	-	-	-	-	-	-	Val180*	-	-	-	-	-	-
-	-	-	-	-	Ile203	-	-	-	-	-	-	-	Ile203	-
-	-	Leu204	-	-	-	-	-	-	-	-	-	-	-	-
-	#Gly205*	-	-	-	Gly205	-	-	-	-	-	-	-	Gly205*	-
#Thr206*	#Thr206*	-	-	-	Thr206	-	-	-	-	-	Thr206	Thr206*	#Thr206*	Thr206*
#His207*	-	-	-	-	-	His207	-	-	His207	His207	His207	-	-	His207
-	-	-	-	Trp230	-	-	#Trp230*	Trp230	Trp230	#Trp230*	#Trp230*	-	-	Trp230
**-17.6525**	**-14.8791**	**-9.5734**	**-8.0396**	**-15.6181**	**-12.0705**	**-10.4349**	**-18.3215**	**-15.653**	**-8.1927**	**-6.7261**	**-6.7703**	**-18.7524**	**-14.3912**	**-19.3639**

* Residues involved in H-bond interactions; #*Residues involved in H-bond and non-bonded interactions. The other residues are involved in non-bonded interactions. Binding energies (kJ/mol) are provided in last row.

Supplementary Table S5. Docking interactions of the binding site residues with all the 15 compounds against 2-cysteine peroxiredoxin (BxPRX) from *B.xylophilus*

**10255**	**25429**	**8663**	**708857**	**42574**	**5430**	**442658**	**4030**	**40854**	**26879**	**3913**	**2871**	**5464102**	**3334**	**35802**
Ile6	-	-	-	-	-	-	Ile6	Ile6	-	-	Ile6	Ile6	Ile6	Ile6
-	-	-	-	Ile6	-	-	-	-	-	-	-	-	-	-
-	-	-	-	-	-	-	-	-	-	-	-	-	-	-
-	-	-	-	-	-	-	-	-	-	-	-	-	-	-
Arg137*	-	-	-	-	-	Arg137*	Arg137*	Arg137*	-	-	-	arg137*	Arg137*	Arg137*
-	-	-	-	#Gln138*	-	#Gln138*	Gln138	Gln138*	-	-	#Gln138*	Gln138	Gln138	#Gln138*
-	-	-	-	#Ile139*	-	-	#Ile139*	-	-	-	Ile139*	ile139*	#Ile139*	#Ile139*
-	Thr140	Thr140	Thr140	-	Thr140	Thr140*	-	-	Thr140	Thr140	-	-	-	-
-	#Asn142*	-	Asn142*	-	-	-	-	-	#Asn142*	-	-	-	-	-
-	Arg148	-	Arg148	-	Arg148	-	-	-	Arg148	Arg148	-	-	-	-
-	Ser149*	Ser149*	#Ser149*	-	#Ser149*	-	-	-	#Ser149*	#Ser149*	-	Leu156	-	-
-	-	-	-	-	-	-	-	-	-	-	-	-	-	-
-	-	Glu152	Glu152	-	Glu152	#Glu152*	-	-	Glu152	Glu152	-	glu152*	-	-
-	-	-	-	-	Thr153	-	-	-	-	#Thr153*	-	-	-	-
-	-	-	-	Arg155*	-	Arg155*	-	-	-	-	Arg155*	-	-	-
-	-	Leu156	-	Leu156	Leu156	Leu156	-	-	-	Leu156	Leu156	-	-	-
-	-	-	-	-	-	-	-	-	-	-	Ala159	-	-	-
Phe160	-	-	-	Phe160	-	Phe160	Phe160	Phe160	-	-	Phe160	Phe160	Phe160	Phe160
**-18.5856**	**-16.525**	**-11.793**	**-12.548**	**-6.0856**	**-15.395**	**-9.6139**	**-20.1114**	**-19.8592**	**-12.361**	**-10.75**	**-18.175**	**-30.1634**	**-18.826**	**-23.2623**

* Residues involved in H-bond interactions; #*Residues involved in H-bond and non-bonded interactions. The other residues are involved in non-bonded interactions. Binding energies (kJ/mol) are provided in last row.

Supplementary Table S6. Docking interactions of the binding site residues with all the 15 compounds against Heat Shock Protein 90 (BxHSP90) from *B.xylophilus*

**10255**	**25429**	**8663**	**708857**	**42574**	**5430**	**442658**	**4030**	**40854**	**26879**	**3913**	**2871**	**5464102**	**3334**	**35802**
-	-	-	-	Trp323	-	-	-	-	#Trp323*	#Trp323*	Trp323*	-	-	-
-	-	-	-	-	-	-	-	-	Met327	-	-	-	-	-
-	-	-	-	-	-	Arg329*	-	-	-	-	-	-	-	-
#Met331*	#Met331*	-	-	Met331	Met331*	-	Met331*	Met331*	Met331	-	Met331	-	Met331*	#Met331*
Lys332	-	Lys332	Lys332	#Lys332*	Lys332	#Lys332*	Lys332	Lys332	-	-	-	#Lys332*	Lys332	Lys332
-	-	-	Ala333	-	-	-	Ala333	Ala333	-	-	-	Ala333	Ala333	-
-	Gln334*	-	#Gln334*	-	Gln334	Gln334*	#Gln334*	Gln334*	-	-	-	Gln334	#Gln334*	#Gln334*
-	-	-	Ala335	-	Ala335	-	Ala335	-	-	-	-	-	Ala335	Ala335
-	-	-	-	-	-	-	-	-	-	-	-	-	-	-
#Arg337*	#Arg337*	#Arg337*	Arg337	Arg337	#Arg337*	-	#Arg337*	#Arg337*	#Arg337*	Arg337*	#Arg337*	#Arg337*	#Arg337*	#Arg337*
Asp338	-	Asp338*	-	Asp338	Asp338	-	-	-	-	-	Asp338	Asp338	-	-
Ser339	-	Ser339	-	Ser339	Ser339	-	-	-	-	-	-	Ser339	-	-
-	-	-	-	-	-	-	-	-	-	-	-	-	-	-
-	-	-	-	-	-	-	-	-	-	Thr341	Thr341	-	-	-
Met342	-	Met342	-	Met342	Met342	Met342	-	-	-	-	-	Met342	-	-
-	-	-	-	-	-	-	-	-	-	-	-	-	-	-
-	-	-	-	#Ser394*	-	#Ser394*	-	-	-	-	-	-	-	-
**-11.942**	**-12.365**	**-9.6124**	**-8.519**	**-8.3846**	**-12.1**	**-4.0356**	**-18.993**	**-13.344**	**-6.1674**	**-5.2535**	**-1.7963**	**-22.895**	**-14.743**	**-15.053**

* Residues involved in H-bond interactions; #*Residues involved in H-bond and non-bonded interactions. The other residues are involved in non-bonded interactions . Binding energies (kJ/mol) are provided in last row.

Supplementary Table S7. Docking interactions of the binding site residues with all the 15 compounds against Venom allergen Protein-3 (BxVAP-3) from *B.xylophilus*

**10255**	**25429**	**8663**	**708857**	**42574**	**5430**	**442658**	**4030**	**40854**	**26879**	**3913**	**2871**	**5464102**	**3334**	**35802**
Tyr79*	-	-	-	Tyr79	Tyr79	-	-	Tyr79	Tyr79	Tyr79	#Tyr79*	-	-	-
-	-	-	-	-	-	-	-	-	-	-	#Leu91*	-	-	-
-	-	-	-	-	-	ala92	-	-	-	-	Ala92	-	-	-
Ala93*	-	#Ala93*	-	#Ala93*	#Ala93*	Ala93*	Ala93	#Ala93*	#Ala93*	#Ala93*	#Ala93*	-	-	ala93
Gln94*	-	Gln94	-	Gln94	Gln94	Gln94	Gln94	Gln94	#Gln94*	Gln94	-	-	-	Gln94
-	#Trp95*	Trp95	#Trp95*	Trp95	-	Trp95	#Trp95*	Trp95	Trp95	-	-	#Trp95*	#Trp95*	#Trp95*
-	-	-	Pro96	-	-	-	-	-	-	-	-	#Pro96*	#Pro96*	-
-	-	-	His97	-	-	-	-	-	-	-	-	His97	#His97*	-
-	-	-	-	-	-	-	-	-	-	-	-	-	Thr98	-
Lys107*	-	-	-	Lys107	-	#Lys107*	-	-	-	-	#Lys107*	-	-	-
-	-	-	-	-	-	-	-	-	-	-	-	-	-	-
-	Asn160	-	-	-	-	-	#Asn160*	Asn160*	-	-	-	#Asn160*	-	#Asn160*
-	-	Trp161	-	Trp161	Trp161	-	Trp161	Trp161	Trp161	Trp161	-	-	-	Trp161
**-12.681**	**-15.173**	**-11.458**	**-9.0794**	**-15.835**	**-13.143**	**-5.3029**	**-18.699**	**-17.071**	**-12.326**	**-12.811**	**-5.8927**	**-19.2792**	**-14.202**	**-17.962**

* Residues involved in H-bond interactions; #*Residues involved in H-bond and non-bonded interactions. The other residues are involved in non-bonded interactions. Binding energies (kJ/mol) are provided in last row.

Supplementary Table S8. Docking interactions of the binding site residues with all the 15 compounds against β-Tubulin (BxTUB) from *B.xylophilus*

**10255**	**25429**	**8663**	**708857**	**42574**	**5430**	**442658**	**4030**	**40854**	**26879**	**3913**	**2871**	**5464102**	**3334**	**35802**
-	Ile6	-	-	-	-	-	-	-	-	-	-	-	-	-
-	-	-	-	Gln11	-	Gln11	Gln11	Gln11*	Gln11	Gln11	Gln11	Gln11	#Gln11*	Gln11*
-	Cys12	-	Cys12	-	Cys12	Cys12	#Cys12*	Cys12	-	-	Cys12	-	Cys12	Cys12
-	-	-	-	-	-	#Gln15*	-	-	-	-	-	-	-	-
-	-	-	-	Glu69	-	-	-	-	-	-	-	-	-	-
-	-	-	-	-	-	-	-	-	Ala97	-	-	-	-	-
-	-	-	-	Gly98*	-	#Gly98*	-	-	Gly98	Gly98	Gly98	Gly98*	-	-
-	-	-	-	#Asn99*	-	#Asn99*	-	#Asn99*	#Asn99*	#Asn99*	#Asn99*	#Asn99*	#Asn99*	#Asn99*
#Ser138*	Ser138*	Ser138	-	-	Ser138*	Ser138*	Ser138	-	-	-	Ser138*	Ser138	#Ser138*	-
Leu139*	-	-	-	-	-	-	-	Gly142*	-	-	-	-	-	-
-	-	-	-	-	Gly140	-	-	-	-	-	-	-	-	-
-	-	-	-	Gly141	-	Gly141	Gly141	Gly141	Gly141	Gly141	Gly141	Gly141	Gly141	Gly141
-	-	-	-	-	-	Gly142	-	-	Gly142*	Gly142*	Gly142*	-	Gly142*	Gly142*
-	-	-	-	-	-	-	-	Thr143*	Thr143*	#Thr143*	Thr143*	Thr143*	Thr143*	Thr143*
Val169	Val169	Val169	Val169	Val169	-	Val169	Val169	Val169	-	-	-	-	Val169	-
Ser172*	-	-	-	-	-	-	Ser172*	-	-	-	-	-	-	-
-	-	-	-	-	-	-	-	-	-	-	--	Ser176	-	-
#Asp177*	Asp177*	#Asp177*	#Asp177*	Asp177	#Asp177*	-	#Asp177*	Asp177	-	-	-	#Asp177*	-	Asp177
-	-	-	Thr178	-	Thr178	-	-	Thr178	--	Thr178	-	-	-	Thr178
#Glu181*	-	#Glu181*	-	-	Glu181	-	-	-	-	-	-	Glu181	-	-
Asn204*	Asn204*	-	Asn204*	-	Asn204*	#Asn204*	Asn204*	-	-	-	-	Asn204	-	Asn204
-	Tyr222	-	Tyr222	-	Tyr222	Tyr222	Tyr222	Tyr222	-	-	-	-	Tyr222	Tyr222
-	-	-	-	-	-	Asn226*	-	-	-	-	-	-	-	-
**-24.909**	**-21.44**	**-12.053**	**-12.559**	**-14.155**	**-16.532**	**-22.876**	**-25.531**	**-21.242**	**-13.724**	**-17.188**	**-15.065**	**-27.122**	**-24.141**	**-28.058**

* Residues involved in H-bond interactions; #*Residues involved in H-bond and non-bonded interactions. The other residues are involved in non-bonded interactions . Binding energies (kJ/mol) are provided in last row.

Supplementary Table S9. DFT calculations pertaining to the molecular chemical reactivity for Amocarzine, flubendazole and Mebendazole

**Compound**	**HOMO**	**LUMO**	**Energy Gap**	**Ionization potential (IP) (eV)**	**Electron affinity (EA) (eV)**	**Electro negativity (χ) (eV)**	**Electro chemical potential (μ) (eV)**	**Hardness (η) (eV)**	**Softness (σ) (eV)**	**Electrophilicity (ω) (eV)**
Amocarzine	-0.9453	-0.3366	0.6087	0.9453	0.3366	0.64095	-0.64095	0.3043	3.2862	-0.6746
Flubendazole	-2.3080	-0.1142	2.1938	2.3080	0.1142	1.2111	-1.2111	1.0969	0.9116	-0.6685
Mebendazole	-2.5317	-0.1904	2.3413	2.5317	0.1904	1.36105	-1.36105	1.1706	0.8542	-0.7912

The calculated HOMO-LUMO orbital energies are used to estimate the ionization energy, electron affinity, electro negativity, electronic chemical potential, molecular hardness, molecular softness, and Electrophilicity index by using the following equations:

Ionization energy (IE) = $-\varepsilon_{HOMO}$,

Electron affinity (EA) = $-\varepsilon_{LUMO}$,

Electro negativity (χ) = (IE + EA)/2,

Electronic chemical potential (μ) = $-\chi$,

Chemical hardness (η) = (IE− EA)/2,

Chemical softness (σ) = $1/\eta$,

Electrophilicity index (ω) = $\mu^2/2\eta$.
